# Atlantoaxial dislocation with congenital “sandwich fusion” in the craniovertebral junction: a retrospective case series of 70 patients

**DOI:** 10.1186/s12891-020-03852-8

**Published:** 2020-12-07

**Authors:** Yinglun Tian, Nanfang Xu, Ming Yan, Peter G. Passias, Frank A. Segreto, Shenglin Wang

**Affiliations:** 1 Department of Orthopaedics, Peking University Third Hospital, No 49 North Garden Street, HaiDian District, Beijing, 100191 People’s Republic of China; 2 Department of Orthopedics, NYU Langone Orthopedic Hospital, New York, NY USA

**Keywords:** Atlantoaxial dislocation, C1 occipitalization, C2–3 fusion, Clinical features, Surgical treatment

## Abstract

**Background:**

In the setting of congenital C1 occipitalization and C2–3 fusion, significant strain is placed on the atlantoaxial joint. Vertebral fusion both above and below the atlantoaxial joint (i.e., a “sandwich”) creates substantial instability. We retrospectively report on a case series of “sandwich fusion” atlantoaxial dislocation (AAD), describing the associated clinical characteristics and detailing surgical treatment. To the best of our knowledge, the present study is the largest investigation to date of this congenital subgroup of AAD.

**Methods:**

Seventy consecutive patients with sandwich fusion AAD, from one senior surgeon, were retrospectively reviewed. The clinical features and the surgical treatment results were assessed using descriptive statistics. No funding sources or potential conflict of interest-associated biases exist.

**Results:**

The mean patient age was 42.2 years (range: 5–77 years); 36 patients were male, and 34 were female. Fifty-eight patients (82.9%) had myelopathy, with Japanese Orthopaedic Association (JOA) scores ranging 4–16 (mean: 12.9). Cranial neuropathy was involved in 10 cases (14.3%). The most common presentation age group was 31 to 40 years (24 cases, 34.3%). Radiological findings revealed brainstem and/or cervical-medullar compression (58 cases, 82.9%), syringomyelia (16 cases, 22.9%), Chiari malformation (12 cases, 17.1%), cervical spinal stenosis (10 cases, 14.3%), high scapula deformity (1 case, 1.4%), os odontoideum (1 case, 1.4%), and dysplasia of the atlas (1 case, 1.4%). Computed tomography angiography was performed in 27 cases, and vertebral artery (VA) anomalies were identified in 14 cases (51.9%). All 70 patients underwent surgical treatment, without spinal cord or VA injury. Four patients (5.7%) suffered complications, including 1 wound infection, 1 screw loosening, and 2 cases of bulbar paralysis. In the 58 patients with myelopathy, the mean JOA score increased from 12.9 to 14.5. The average follow-up time was 50.5 months (range: 24–120 months). All 70 cases achieved solid atlantoaxial fusion at the final follow-up.

**Conclusions:**

Sandwich fusion AAD, a unique subgroup of AAD, has distinctive clinical features and associated malformations such as cervical-medullar compression, syringomyelia, and VA anomalies. Surgical treatment of AAD was associated with myelopathy improvement and minimal complication occurrence.

## Background

**Fig. 1:**
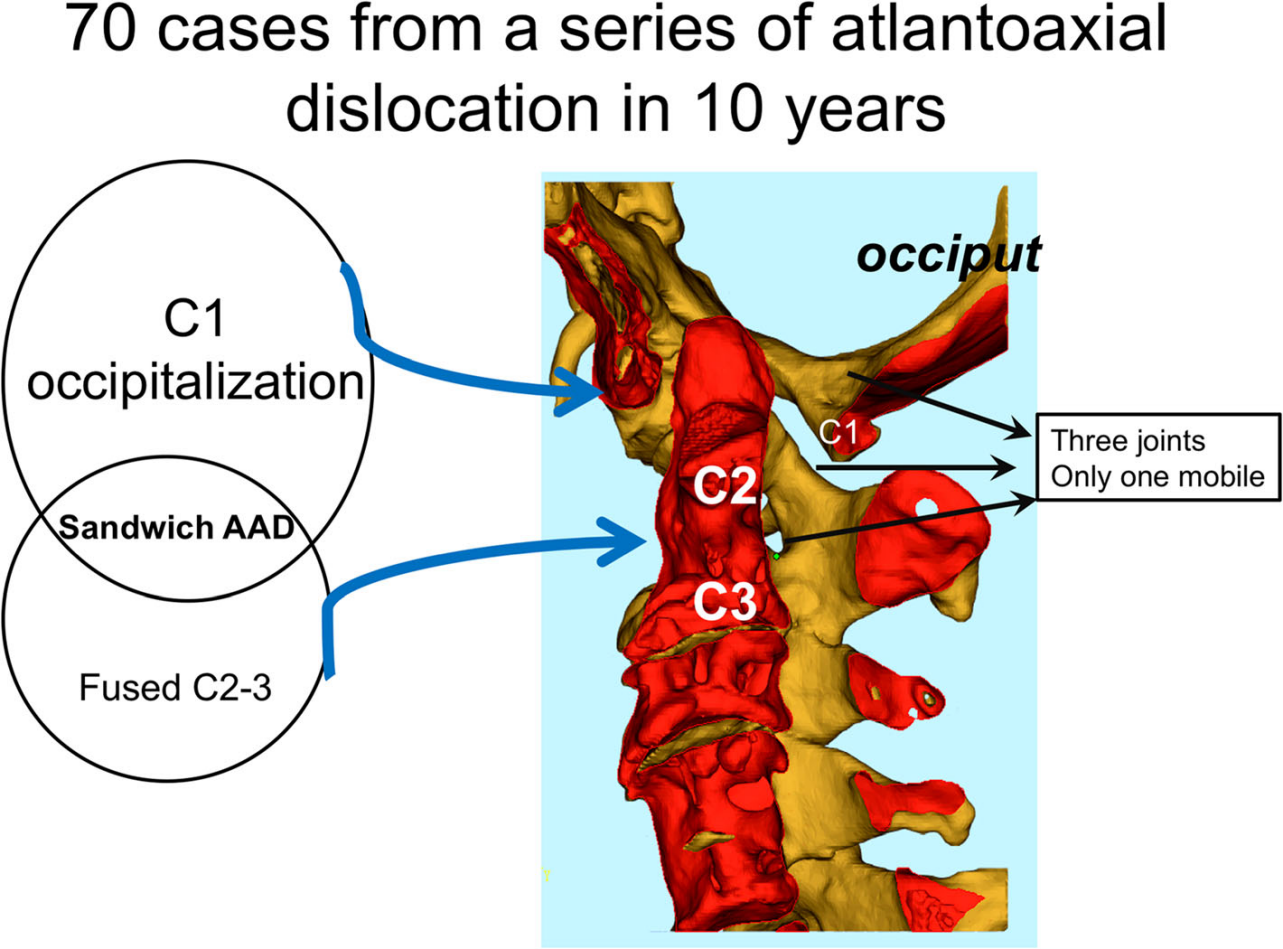
A subgroup from AAD: sandwich mechanism

Atlantoaxial dislocation (AAD) is often associated with complex deformities of the craniovertebral junction and poses a significant risk of neurological deterioration [1, 2]. With regards to its etiology, AAD can be divided into three main categories: traumatic, idiopathic, and deformity-related. Among those individuals with congenital or developmental deformities, the concomitance of C1 occipitalization and C2–3 fusion constitutes a subgroup with a particularly high risk of AAD development. In the current study, we refer to the condition of C1 occipitalization, C2–3 congenital fusion, and subsequent AAD as AAD with “sandwich fusion” (Fig. 1).

This condition results in an anterior atlanto-dental interval (ADI) and resultant AAD, with superior odontoid migration and hypermobility of the craniovertebral junction, and resultant symptomatic stenosis [2]. Previously, 47–68% of patients with occipitalization have been reported to have concurrent C2–3 fusion [3]. Congenital C2–3 fusions (block C2–3 vertebrae) typically result in increased stress of the adjacent vertebral segments, compounding the inherent risk of AAD among C1 occipitalization cases. A previous study reported that 57% of isolated congenital C1 occipitalization cases went on to develop subsequent AAD [4].

In the context of sandwich fusion, significant strain is placed on the atlantoaxial joint. Resulting instability develops in the atlantoaxial joint, which is juxtaposed between fusion occurring both above and below the joint (it is “sandwiched”), and normal anatomical relationships are disrupted. To overcome this biomechanical limitation, the authors have previously developed a novel standard instrumentation technique, which uses a C3 lateral mass screw (C3LMS) and a C2 pedicle screw [5].

An additional challenge in the surgical treatment of sandwich fusion AAD patients is the strong association of the condition with vascular anomalies [6–8]. Performing preoperative computed tomography (CT) angiography is recommended for surgical planning, in order to identify vascular anomalies and minimize the risk of injury to the vertebral arteries [6, 9].

Additionally, the clinical spectrum and natural history of atlas occipitalization have not been clearly defined. Occipitalization can range from being an asymptomatic condition to one that causes substantial pain and varying degrees of neurological deficit, including progressive cervical myelopathy [10]. Symptoms may also be delayed and not develop until the third or fourth decade of life [3, 11, 12]. One reason for the late presentation of clinical symptoms is that the associated atlantoaxial instability progresses with age, resulting in gradual spinal cord and/or vertebral artery (VA) compromise. Regarding sandwich fusion AAD, the natural history and the efficacy of surgical management are poorly understood [13]. We hypothesize that sandwich fusion AAD patients become symptomatic at a younger age, progress in terms of severity at a more rapid rate, and potentially achieve inferior treatment outcomes from surgical intervention compared to typical AAD presentations.

## Methods

### Study design

A retrospective chart review of consecutive sandwich fusion AAD cases was performed at a single institution during a period of 10 years (from September 2008 to July 2019).

### Patient recruitment

Institutional Review Board approval was obtained before the initiation of this study. The inclusion criterion was a diagnosis of AAD with concomitant C1 occipitalization and C2–3 congenital fusion. The diagnosis of AAD was based on dynamic radiographs (flexion and extension) and CT scan evidence. AAD was defined as an abnormal local relationship between the atlas and axis, with an ADI > 3 mm in adults and > 5 mm in children (< 18 years of age). The ADI was measured from the anterior arch of the atlas to the odontoid process. Patients were excluded if they did not undergo surgical treatment, or if adequate radiographs and clinical follow-up information were unavailable.

### Surgical management

**Fig. 2:**
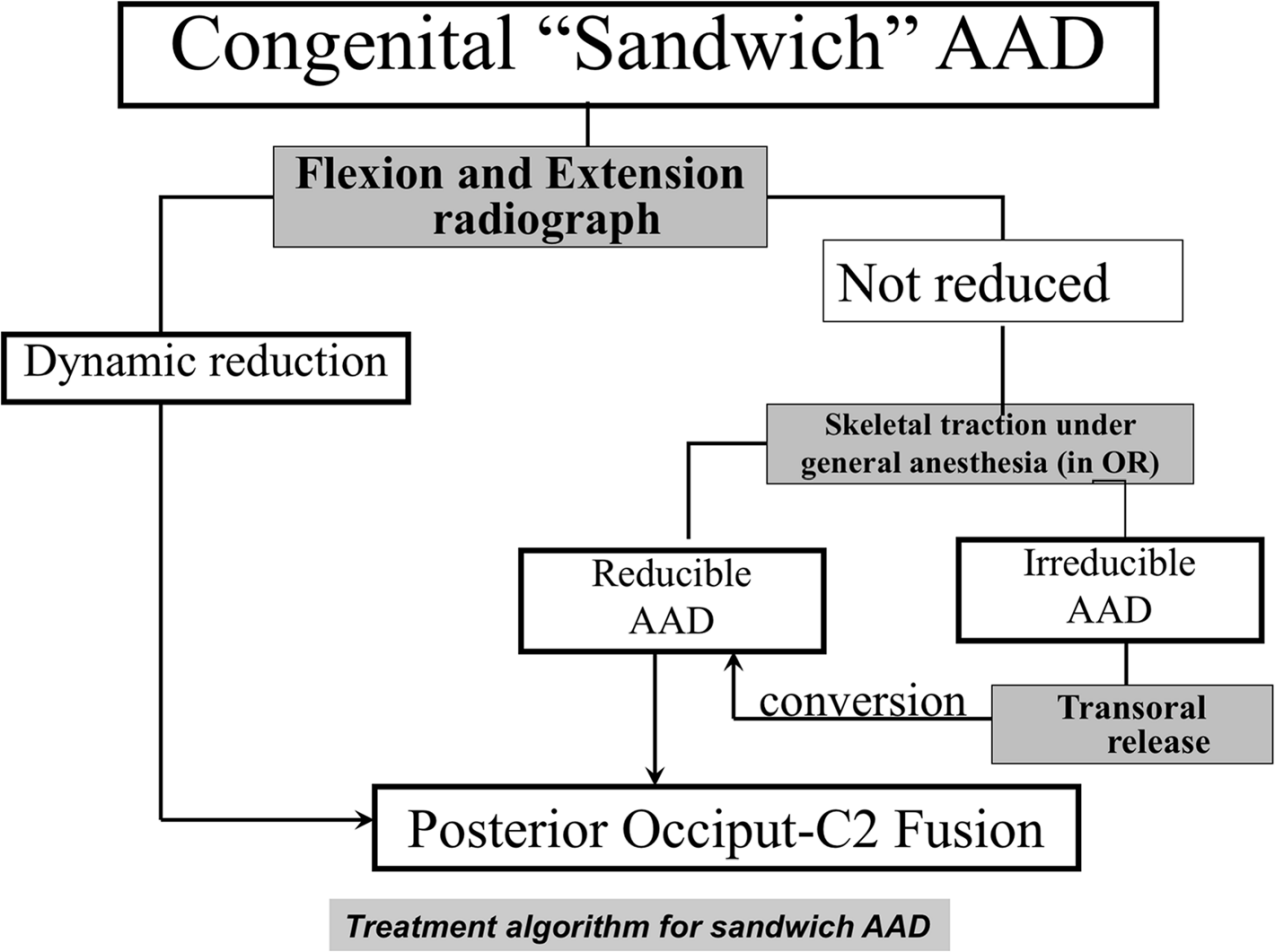
Treatment algorithm

All patients included in this series underwent surgical management. Reducibility was defined by using a previously published protocol, incorporating dynamic radiographic imaging, CT imaging, and an intraoperative traction test when indicated [14, 15]. If the atlantoaxial joint was reducible [15], isolated posterior fixation and fusion were performed using one of the following techniques: occiput-to-C2 fixation, occiput-to-C2 and -C3 fixation, or occiput-to-C3 and -C4 fixation. The cervical fixation included pedicle, laminar, and lateral mass screws. If the C2 pedicles were sufficiently developed to accommodate pedicle screws, bilateral occiput-to-C2 fixation was our first choice technique. Otherwise, C2 laminar screws were used as a salvage procedure. If the C2 fixation procedure failed, or the C2 pedicle screws were not reliable, the adjacent C3 or even C4 pedicles could be selected to conduct the occipital-cervical fixation. If the atlantoaxial joint was irreducible, the patients first underwent transoral atlantoaxial release. Following this, the posterior approach was performed. Our treatment algorithm is summarized in Fig. 2.

Following the installation of instrumentation, the occiput, C2 lamina, and C2 spinous process were decorticated with a high-speed burr. Morselized cancellous grafts harvested from the posterior iliac crest were bridged between the occiput and the C2 lamina. Collars or bracing were not typically indicated postoperatively, although they were utilized in cases where severe dislocation and viscoelastic rebound forces were noted during the procedure; in such cases, an additional halo-vest was used postoperatively for supplemental stability. This regimen was continued until arthrodesis was achieved (assessed by a 3-month CT scan).

### Statistical analysis

All radiographic findings, clinical characteristics, surgical details, and treatment outcomes were analyzed using frequencies and descriptive statistics.

## Results

### Clinical presentation

**Fig. 3:**
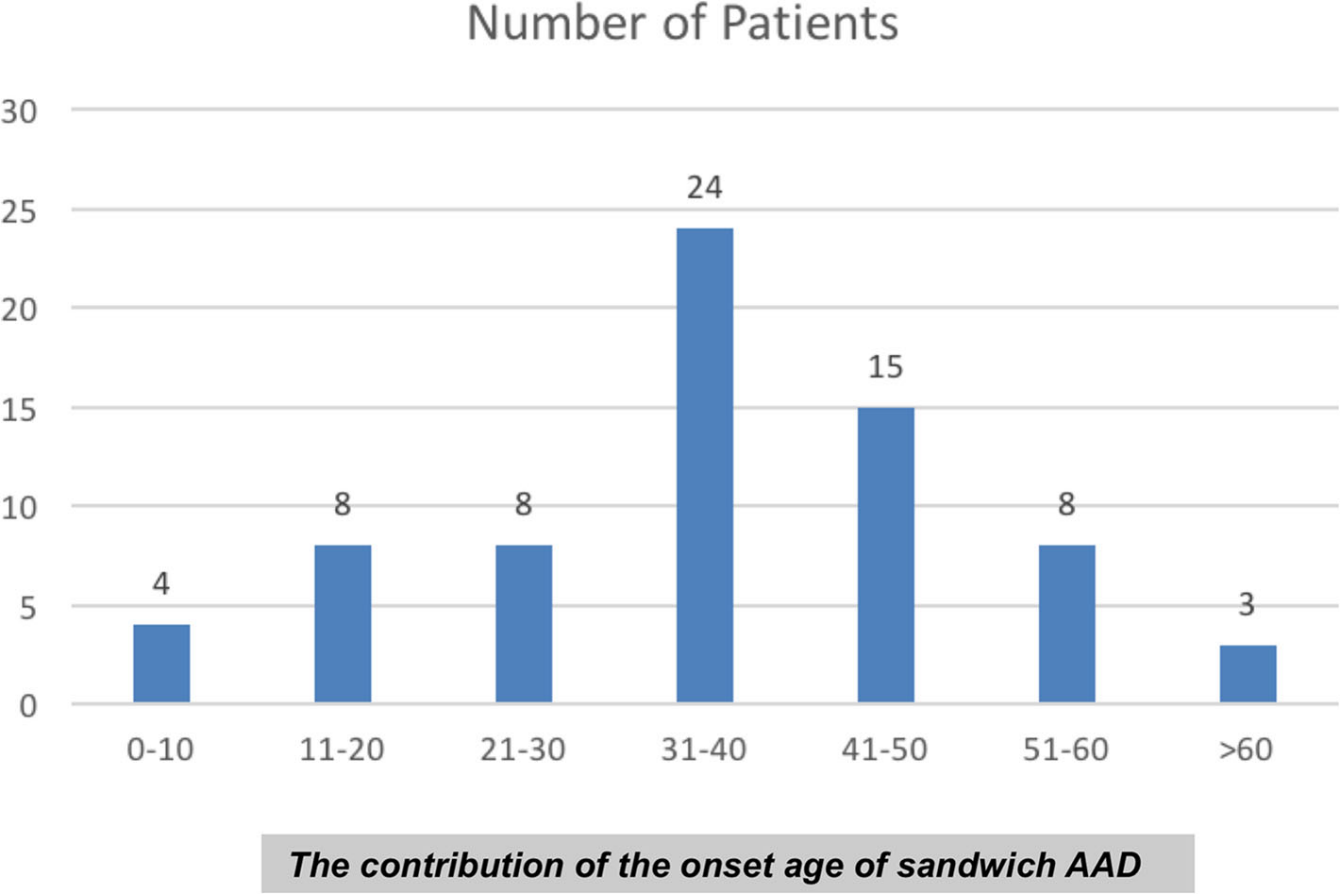
The contribution of onset age

Seventy patients were diagnosed as having AAD with concomitant C1 occipitalization and C2–3 congenital fusion (sandwich fusion AAD). The mean age at the initial visit was 42.2 years (range: 5–77 years); 36 patients were male and 34 were female. The mean age of patient presentation was 36.5 years. The onset/presentation age is summarized in Fig. 3. The duration of clinical symptoms prior to operative treatment averaged 111.2 months, and symptoms included weakness, numbness, and clumsiness of the limbs, unstable gait, cranial nerve dysfunction, torticollis, neck pain or restriction, discomfort of the shoulders, sphincter disturbances, and vertigo (Table 1). The most common symptoms were weakness, numbness, and clumsiness of the limbs (54 cases, 77.1%), unstable gait (30 cases, 42.9%), and vertigo (20 cases, 28.6%). Further clinical presentation details can be found in Table 1. Fifty-eight patients (82.9%) also presented with myelopathy, with Japanese Orthopaedic Association (JOA) scores (the most commonly used evaluation tool for neurological function among patients with cervical myelopathy) ranging from 4 to 16 (mean: 12.9). The JOA scoring system has been reported to have excellent reliability for patients with cervical myelopathy [16]. This scale is composed of six domain scores (motor dysfunction in the upper extremities, motor dysfunction in the lower extremities, sensory function in the upper extremities, sensory function in the trunk, sensory function in the lower extremities, and bladder function), each scaled from 0 to 4, 4, 2, 2, 2, and 3, respectively, with the minimum total score being 0 and the maximum total score being 17 [17]. Ten cases involved symptoms of cranial neuropathy, including dysphagia (9 cases), dysarthria (3 cases), and nystagmus (3 cases).

**Table 1:** Clinical Presentations of the Patients

Symptom and Signs	Patients, n	Percentage %
Weakness, numb and/or clumsiness of limbs	54	77.1
Unstable gait	30	42.9
vertigo	20	28.6
Cranial nerve dysfunction	9	12.9
Discomfort of the shoulders	7	10.0
Neck pain or neck restriction	14	20.0
Torticollis	4	5.7
sphincter disturbances	2	2.9
Total	70	100

### Radiological findings

All patients had sandwich fusion deformities, including the concurrent presence of C1 occipitalization, C2–3 congenital fusion, and AAD. Three patients also had additional congenital spinal fusions: C5 and C6 (1 case), C2–3 and C6–7 (1 case), and T3–6 (1 case). Preoperatively, ADI ranged from 2.43 mm to 12.79 mm (mean: 5.98 mm), with the postoperative ADI ranging from 0 mm to 7.8 mm (mean: 2.28 mm). The mean distances by which the odontoid exceeded the Chamberlain line preoperatively and postoperatively were 9.01 mm (range: 0–19.34 mm) and 5.66 mm (range: 0–17.97 mm), respectively. The initial cervical-medullary angle ranged from 106.9° to 164° (mean: 134.6°), with the postoperative cervical-medullary angle ranging from 115° to 166° (mean: 141.5°). Associated radiographic malformations included brainstem and/or cervical-medullar compression (58 cases, 82.9%), syringomyelia (16 cases, 22.9%), Chiari malformation (12 cases, 17.1%), cervical spinal stenosis (10 cases, 14.3%), high scapula deformity (1 case, 1.4%), os odontoideum (OO) (1 case, 1.4%), and dysplasia of the atlas (1 case, 1.4%).

**Fig. 4:**
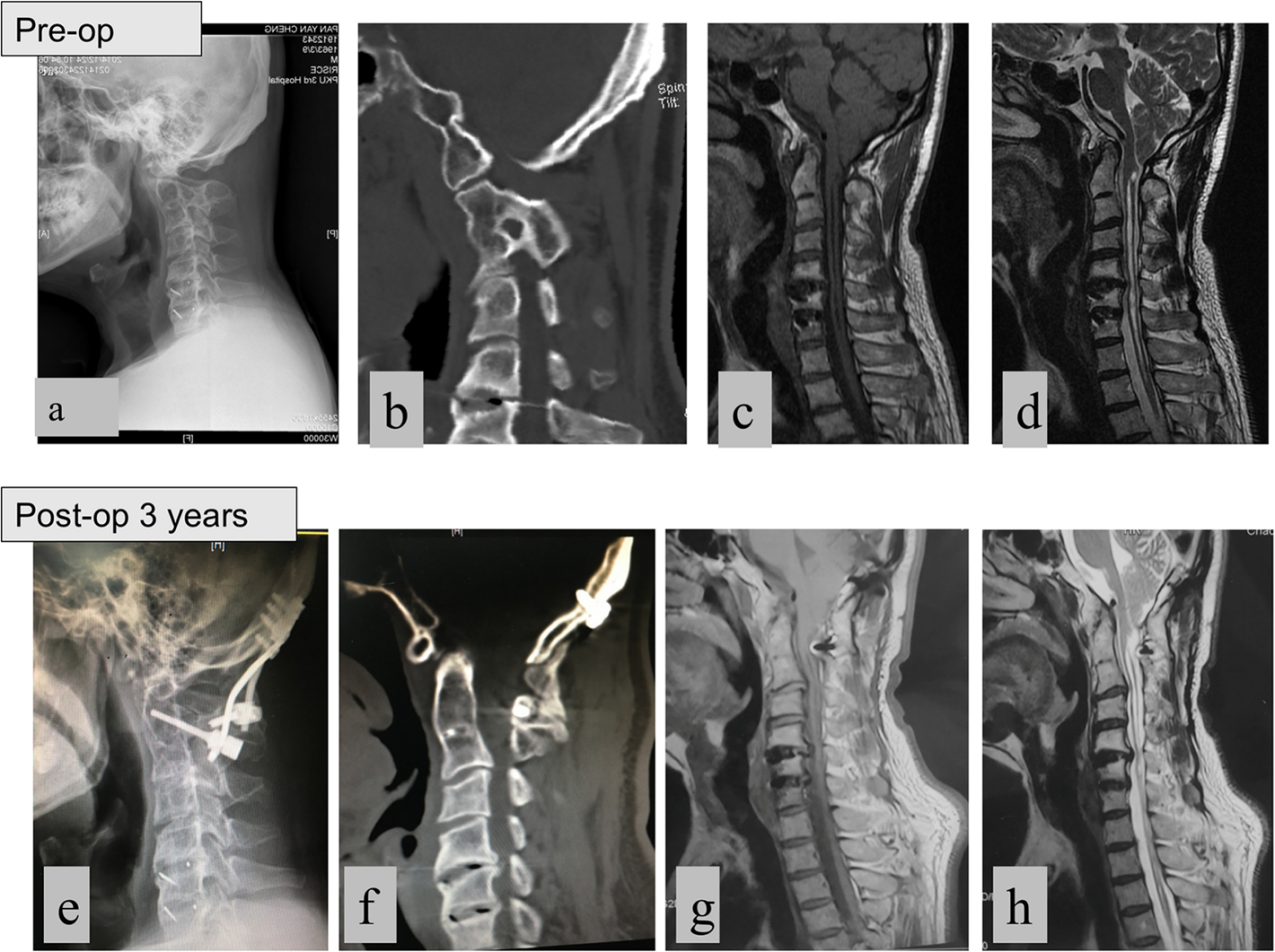
A 51-y/o male patient with severe myelopathy. **a** lateral radiograph showed sandwich AAD, and ADI = 5.5 mm; **b** CT indicated C1–2 facet remodeling (sloped from horizontally to vertically); **c** and **d** MRI showed Chiari deformity and syringomyelia from C2 to T2; **e** C2 laminar screw and pedicle screw fixation on 3-year follow-up radiograph (lateral); **f** 3-year follow-up CT showed the bony fusion between the occiput and the axis; **g** and **h**: 3-year follow-up MRI showed the partial relief of Chiari deformity and syringomyelia

As demonstrated in Fig. 4, the case of a 51-year-old man with preoperative radiographs and CT imaging revealed sandwich fusion AAD, odontoid migration, and remodeling of the facets (sloped from horizontally to vertically, Fig. 4b). Magnetic resonance imaging (MRI) revealed severe compression of the cervical-medullary junction by upward displacement of the odontoid, tonsillar herniation dropping to the C2 level, a large syringomyelia extending from the C2 to the T2 level, and canal stenosis at the C3–4 and C5–6 levels (Fig. 4).

**Fig. 5:**
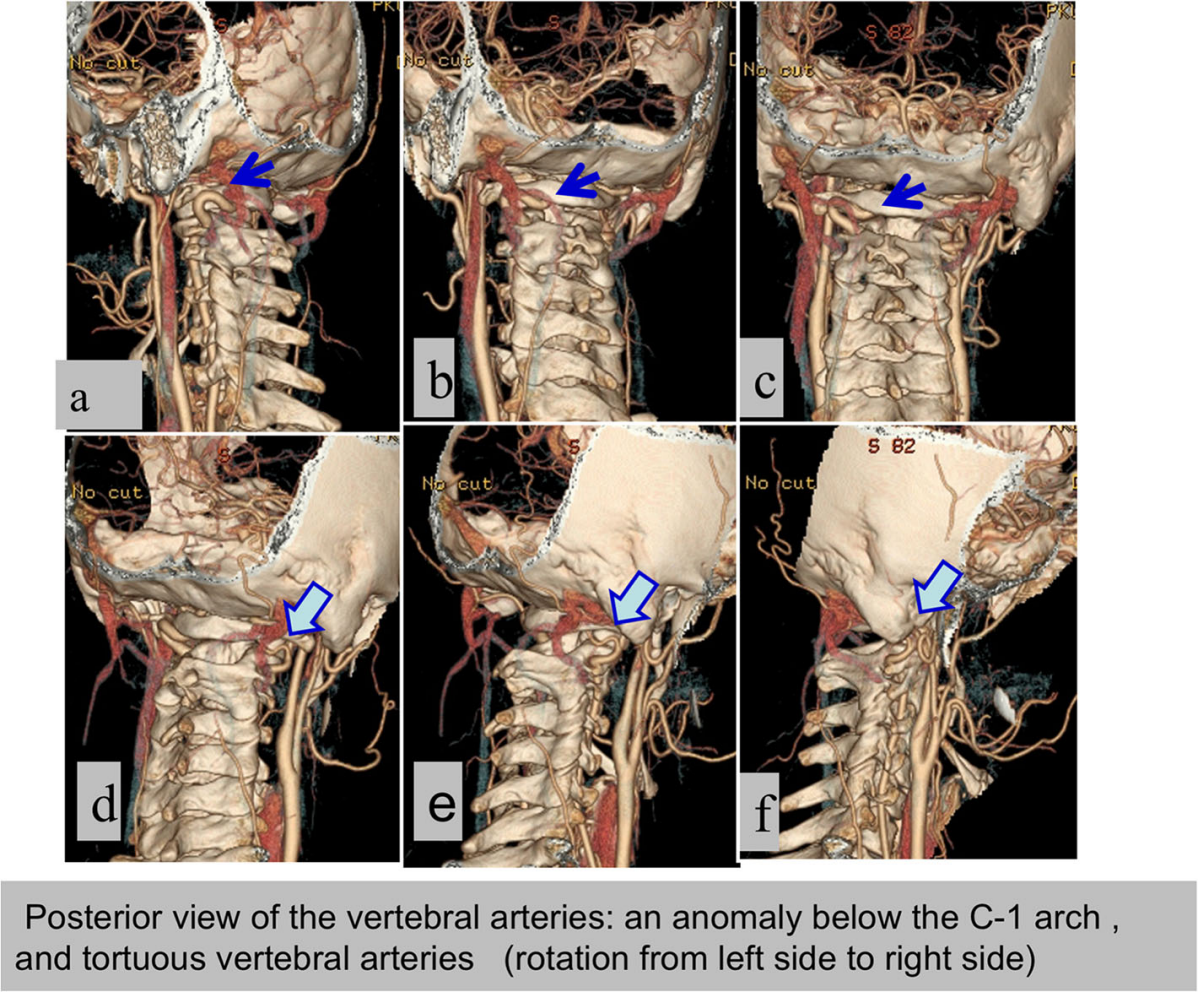
Anomaly of vertebral arteries on CTA. **a, b** and **c**: the left lateral, left posterior oblique and posterior view of the VA; the blue arrow showed left VA was tortuous and anomalous riding under the C1 arch. **d**,**e**&**f**: the right lateral view of the VA; the vacant arrow showed right VA was also tortuous under the C1 arch

From 2013, CT angiography was performed in 27 cases. Among these, VA anomalies were identified in 14 cases (51.9%), including VA hypoplasia (10 sides in 9 cases), tortuous VAs (9 sides in 5 cases), an anomaly below the C1 arch (8 sides in 5 cases) (Fig. 5), “high-riding” VAs invading the C2 pedicle (2 sides in 2 cases), VA aplasia (1 side in 1 case), and fenestration of the VA (1 side in 1 case).

### Surgical summary

**Fig. 6:**
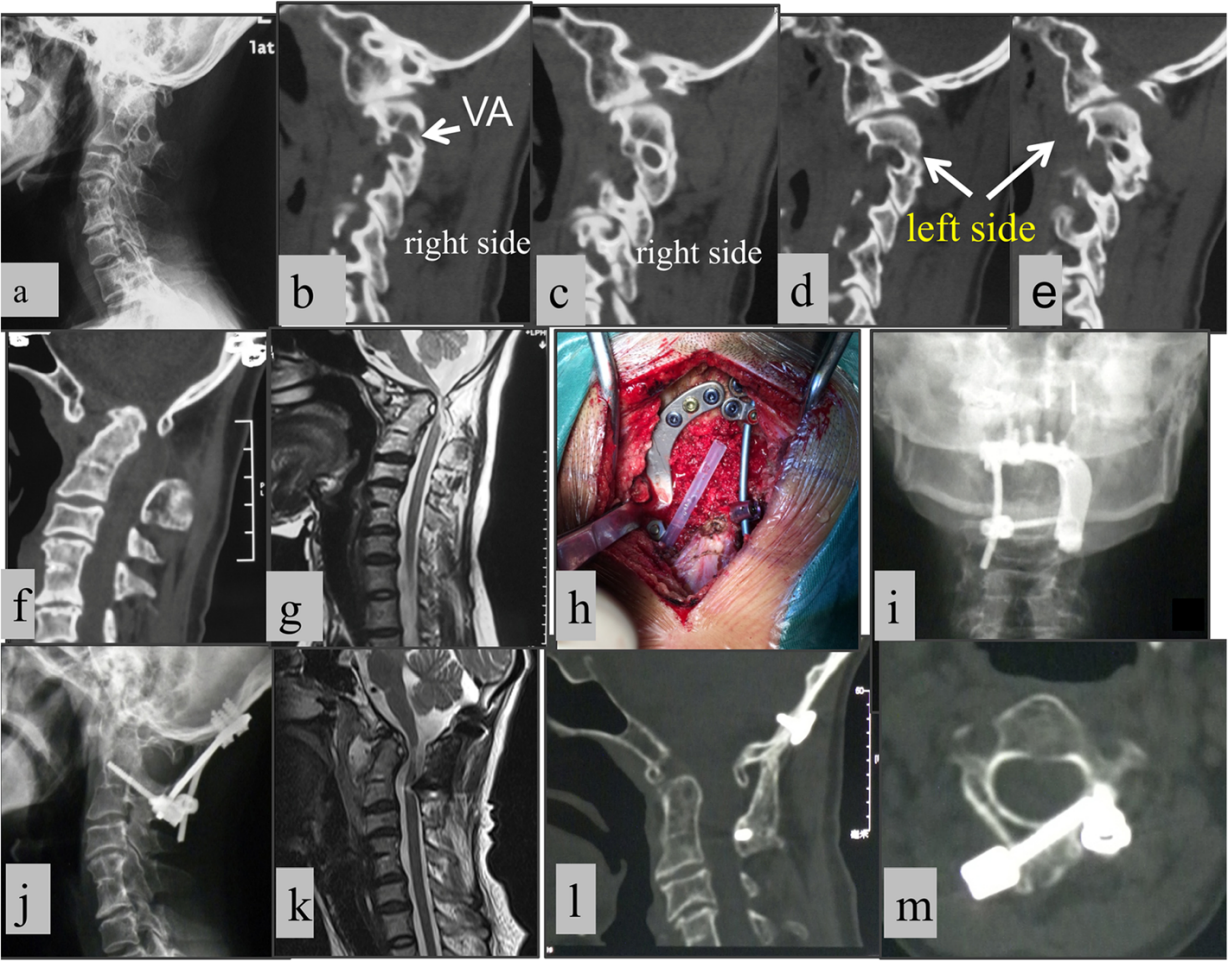
**a** The pre-operative radiographs of a 44-year-old woman showed sandwich AAD, C1 occipitalization and C2–3 and C5–6 congenital fusion. **b-d** Preoperative CT revealed the left C2 pedicle can accommodate a 3.5-mm screw (**c**), while the right pedicle was “thin”and occupied by VA. **e**. The odontoid invaded the foramen magnum, and ADI was abnormal (10 mm). **f**. The pre-operative mid-sagittal CT scan. **g**. The pre-operative MRI showed severe compression of the spinal cord. **h**. During the surgery: On the left side, C2PS was plated and connected with the occiput by a plate; On the right side, C2LS was inserted and connected with the occiput screws by a rod. Morselizedcancellous grafts harvested from the posterior iliac crest were bridged between the occiput and C2 lamina. **i**. The post-operative radiographs (AP). **j**. The post-operative radiographs (Lateral). **k**. The post-operative MRI showed adequate decompression. **l**. Postoperative CT (after 4 months) confirmed complete reduction and rigid fusion between occiput and C2. **m**. The postoperative CT showed the techniques included C2PS and C2LS

All 70 patients underwent surgical treatment (Fig. 6). The surgery was either posterior occipitocervical fusion (reducible dislocation, 55 cases) or transoral release followed by posterior fusion in the same setting (irreducible dislocation, 15 cases). Of the 70 cases, 46 involved posterior lower instrumentation to vertebra C2; 13, to C2–3; and 3, to C1–2 (Table 2). The estimated blood loss ranged from 50 mL to 500 mL (mean: 177.1 mL).

**Table 2:** Operation Methods

Fixed segments	Patients, n
C0-C2	46
C0-C23	13
C0-C12	3
C0-C234	2
C1-C2	2
C0-C3	1
C0-C123	1
C0-C245	1
C0-C345	1
Total	70

Of the 70 patients, 26 underwent posterior asymmetrical and mixed fixations, including 21 using two different techniques (Fig. 6), 4 using three different techniques, and 1 using four different techniques. The reasons for mixed fixations were a high-riding VA at the C2 segment, pedicle or vertebral hypogenesis at C2, and blocked and hypoplastic pedicles in a patient with Klippel Feil syndrome, respectively.

### Postoperative outcomes

The average follow-up time was 50.5 months (range: 24–120 months). The overall complication rate was 5.7% (4 patients). No spinal cord or VA injuries were observed. Rather, the complications were an incision infection (1 patient, underwent debridement), screw loosening with recurrent dislocation (1 patient, underwent revision), and bulbar paralysis with severe dysphagia (2 patients, self-cured). The patient who experienced screw loosening originally underwent combined fixation with a C2 pedicle screw and a C2 lateral mass screw, with complete atlantoaxial reduction. His myelopathy improved during the first 3 months postoperatively but deteriorated later with the screws loosening and recurrent dislocation occurring. At this point, transoral revision was performed and the odontoid was removed. Myelopathy improved and was superior to the preoperative situation.

Among the 58 patients with myelopathy, neurological improvement was observed in 13 (72.4%), for whom motor, sensory, and bladder function improved to varying degrees. We usually use minimum detectable change (MDC) or minimum clinically important difference (MCID) to evaluate the psychometric properties of the JOA score. The MDC and MCID for JOA scores in patients with cervical myelopathy have been calculated to be 1.25. Therefore, when the JOA score increased by more than 1.25, we considered it to represent a clinical improvement [18]. Correspondingly, the postoperative JOA scores ranged from 5 to 17 (mean: 14.5), which represented an increase from a range of 4 to 16 (mean: 12.9) preoperatively. All 70 patients achieved solid atlantoaxial fusion at final follow-up (Fig. 6). Among 13 of the 16 syringomyelia patients who underwent MRI during follow-up, the syringomyelia disappeared in 5 patients, decreased in 5 patients, and remained unchanged in 3 patients.

## Discussion

Within the literature, sandwich fusion has previously been described in several small series. A study of 33 AAD patients by Shen at al [2] reported that 13 of the 33 cases exhibited occipitalization and C2–3 fused block vertebra. In 2007, Gholve et al. [3] reviewed all cases of occipitalization from the Children’s Hospital of Philadelphia, 14 of which could be diagnosed with sandwich fusion AAD.

### Pathogenesis of multiple associated malformations

Some scholars believe that atlas occipitalization and congenital C2–3 fusion often lead to AAD and superior odontoid migration, eventually resulting in hypermobility or symptomatic stenosis of the craniovertebral junction [2, 19]. In a study by Gholve et al. [3], AAD was concomitant with occipitalization in 57% of cases. Severity at presentation has also been shown to be distinct in such cases. A study by Shen et al. also found that patients with occipitalization and congenital C2–3 fusion tended to have greater anterior ADI values compared to non-sandwich fusion AAD patients. Sandwich fusion AAD patients often have cervical myelopathy or concomitant anomalies such as basilar invagination, secondary Chiari I malformation, and syringomyelia [3, 20, 21], which typically progress to require occipitocervical reduction and fixation. However, not all sandwich fusion AAD patients express the complete range of clinical features. In our series, the most common concomitant deformities were myelopathy (58 cases, 82.9%), syringomyelia (16 cases, 22.9%), and Chiari malformation (12 cases, 17.1%).

For sandwich fusion AAD patients within the present study, since the fused segments had a decreased range of motion (ROM), we hypothesize that the ROM between C1 and C2 increased in compensation, leading to further stress between C1 and C2, similar to a clamp. The concomitant odontoid migration, basilar invagination, Chiari I malformation, cranial neuropathy, and syringomyelia are thought to be secondary pathologies. The multiple malformations interact, ultimately resulting in the symptoms of cervical stiffness and pain, myelopathy, and lower cranial neuropathy.

### Clinical characteristics of sandwich fusion AAD

Comparing the age, symptoms, and clinical outcomes at the time of presentation in our sandwich fusion cohort with previous reports from a large case series of typical AAD patients [15] and patients exclusively with OO [22], we found that mean presentation age of our patients was 36.5 years, while the typical AAD [15] and OO patients [22] presented at 37.0 and 38.6 years of age, respectively. The most common presentation age group was 31 to 40 years (24 cases, 34.3%), which is younger than that of the typical AAD series (40–49 years) and that of the OO series (41–50 years). Although the sample size of the present study (70 cases) is lower than the typical AAD series (904 cases) and the OO series (279 cases), we hypothesize that sandwich fusion AAD patients intuitively present at a younger age than typical AAD patients because of increased biomechanical stress on the atlantoaxial joint.

Sandwich fusion AAD patients within our cohort had multiple clinical presentations, with the most frequent symptoms and signs including myelopathy, neck and/or occipital pain, and lower cranial neuropathy. Notably, 58 cases (82.9%) involved myelopathy, which is higher than previous reports for typical AAD (66.5%, 601/904) [6]. Also notable was the neurological recovery rate, with 72.4% of patients showing neurological improvement within our sandwich fusion group, lower than the historical recovery rate among typical AAD patients (84.1%) [15].

In the typical AAD series with 904 cases, the incidence of Chiari deformity and syringomyelia was 5.7% (52/904) and 7.2% (65/904) [15], respectively. Sandwich fusion AAD patients within our study presented with substantially higher rates of Chiari deformity (17.1%) and syringomyelia (22.9%). Consequently, a considerable number of patients (10/70, 14.3%) within our sandwich fusion group suffered lower cranial neuropathy.

### Treatment consideration and pitfalls

Patients with congenital C2–3 fusion tend to have thinner C2 pedicles than those without congenital fusion [23]. It has also been previously indicated that 39.6% of patients with congenital C2–3 fusion have inadequate C2 pedicles for a 3.5-mm diameter screw [24]. Additionally, anomalous VAs potentially influence the procedures of craniovertebral realignment and posterior fixation. Twenty-seven patients in the present series underwent CT angiography (representing the largest cohort of sandwich fusion patients undergoing CT angiography in the literature), and 14 out of the 27 patients (51.9%) were found to demonstrate various forms of VA anomaly, including VA hypoplasia/aplasia, tortuous VA, an anomaly below the C1 arch, and high-riding VA. This prevalence rate is higher than those rates previously associated with other types of deformities in the cranio-cervical region [6, 25, 26]. Due to the presence of significant VA and osseous anomalies, we failed to place C2 or C3 pedicle screws in 21 cases within our sandwich fusion cohort and transitioned to salvage techniques. However, these salvage techniques were not always successful due to the increased stress between C1 and C2 in the setting of sandwich fusion and given the limited purchasing power that is intrinsic to trans-laminar screws, as demonstrated in one patient with instrumentation failure who required a subsequent revision procedure. Accordingly, in patients with significant anterior displacement of C1, either anterior or posterior release of the C1/2 joints may be recommended to decrease the stress on posterior implants and to prevent instrumentation failure [27].

Given the findings of the present series, in combination with the literature [28, 29], we now recommend that prior to operating on a patient with sandwich fusion AAD, imaging of the VA should be scrutinized for anomalies, and detailed surgical planning of the procedures and potential salvage techniques should be done.

#### Limitations

We acknowledge several limitations that can form the basis for future investigation. First, we did not systematically compare clinical outcomes between various age groups. Second, the average follow-up time was limited, at 50.5 months; further long-term follow-up data are required to assess the outcomes and durability of our treatment strategy. Third, we did not use the JOA Cervical Myelopathy Evaluation Questionnaire to evaluate cervical myelopathy, which may be a more comprehensive mode of evaluation than the JOA score, since it includes patient satisfaction, disability, handicaps, and general health [30].

## Conclusion

This report evaluated 70 patients with sandwich fusion AAD who underwent surgical management. Within this series, sandwich fusion AAD patients presented at a relatively younger age, with an increased probability of concomitant Chiari deformity, syringomyelia, and cranial neuropathy, normal rates of VA anomalies, and a lower rate of myelopathy improvement compared to patients with typical AAD. Due to substantial osseous and vascular anomalies, careful VA scrutiny and detailed planning of surgical procedures and salvage techniques should be utilized in the treatment of patients with sandwich fusion AAD.

## Data Availability

This study is based on confidential patient data which are available upon request from the author SLW.

## Abbreviations

AAD: Atlantoaxial Dislocation
VA: Vertebral Artery
C3LMS: C3 Lateral Mass Screw
ADI: Atlanto-dental Interval
OO: Os Odontoideum
MDC: Minimum Detectable Change
MCID: Minimum Clinically Important Difference
JOA: Japanese Orthopaedic Association
CT: Computed Tomography
MRI: Magnetic Resonance Imaging
ROM: Range of Motion
